# A *Physarum*-inspired approach to the Euclidean Steiner tree problem

**DOI:** 10.1038/s41598-022-18316-3

**Published:** 2022-08-25

**Authors:** Sheryl Hsu, Fidel I. Schaposnik Massolo, Laura P. Schaposnik

**Affiliations:** 1 Valley Christian High School, San Jose, USA; 2 Institut des Hautes Etudes Scientifiques, Bures-sur-Yvette, France; 3 University of Illinois, Chicago, USA

**Keywords:** Theoretical ecology, Applied microbiology, Cellular microbiology, Applied mathematics, Computer science, Biological physics

## Abstract

This paper presents a novel biologically-inspired explore-and-fuse approach to solving a large array of problems. The inspiration comes from Physarum, a unicellular slime mold capable of solving the traveling salesman and Steiner tree problems. Besides exhibiting individual intelligence, *Physarum* can also share information with other *Physarum* organisms through fusion. These characteristics of Physarum imply that spawning many such organisms we can explore the problem space in parallel, each individual gathering information and forming partial solutions pertaining to a local region of the problem space. When the organisms meet, they fuse and share information, eventually forming one organism which has a global view of the problem and can apply its intelligence to find an overall solution to the problem. This approach can be seen as a “softer” method of divide and conquer. We demonstrate this novel approach, developing the *Physarum Steiner Algorithm* which is capable of finding feasible solutions to the Euclidean Steiner tree problem. This algorithm is of particular interest due to its resemblance to *Physarum polycephalum*, ability to leverage parallel processing, avoid obstacles, and operate on various shapes and topological surfaces including the rectilinear grid.

## Introduction

As the scale and complexity of real-world problems continues to grow, new approaches are increasingly needed to effectively solve them. For example, in 2020, the number of packages shipped exceeded 131 billion and this volume is forecasted to more than double by 2026, putting increased pressure on our ability to efficiently route packages^1^.

A widely-used approach to solving large problems is divide-and-conquer. In the divide-and-conquer paradigm, the problem is recursively partitioned into smaller sub problems. The sub problems are then independently solved and their solutions are combined to form the overall solution to the problem. However, one flaw of divide-and-conquer is that some information cannot be shared between subproblems (although some can, e.g., upper bounds). This can be problematic as oftentimes problems cannot be efficiently divided into independent sub problems.

In this paper, we present *explore-and-fuse*, an alternate, biologically-inspired approach to solving large-scale problems that cannot be broken into independent sub problems, leading in particular to the following contributions:

1. An alternative approach to solving difficult problems. We introduce the *explore-and-fuse* approach and demonstrate its ability to solve difficult problems such as the Steiner tree problem that are not amenable to the traditional divide-and-conquer method.
2. A showcase of the potential of biologically inspired computing. In recent years, there has been an increased effort to develop biologically inspired-computing devices and software programs. Our work highlights the potential for such advancements.

Our approach is inspired by *Physarum*, a unicellular slime mold that can solve mazes, form Steiner trees, solve the traveling salesman problem, and design high-quality networks^2–4,36^. In addition, *Physarum* has the ability to share information with other *Physarum* organisms through fusion^5^, suggesting that we may be able to use multiple *Physarum* to explore a problem in parallel and then leverage their ability to fuse to aggregate the information gained by each of them. In this paper, we use the model of multiple CELLs we introduced in^6^, which is a cellular automaton model of *Physarum* organisms fusing, to form *Physarum* swarms. These swarms are made out of many individual *Physarum* organisms, allowing us to take advantage of its unique features:

- *Physarum* swarms are unique as most swarm algorithms are of more complex animals such as ants or bees while *Physarum* is a single-celled organisms;
- *Physarum* cells are able to solve mazes and form networks;
- *Physarum* cells are able to fuse and share intelligence upon merging.

More specifically, in the *explore-and-fuse* approach, we deploy multiple *Physarum* organisms to independently explore the problem space. As they explore, they meet and fuse, sharing the information that they gathered locally in their exploration. This process continues until the problem space is adequately explored and all the organisms have fused into a single organism that embodies all the local information gathered about the problem. At this point, this organism has a global view of the problem and can proceed to solve the overall problem.

The *explore-and-fuse* paradigm can be seen as a less rigid form of divide-and-conquer. Instead of dividing a problem into independent sub problems, *explore-and-fuse* distributes the problem to multiple organisms for exploration, allowing these organisms to determine the boundaries of their own exploration.

In this set up, these organisms do not conquer or completely solve their sub problems. Instead, they provide partial solutions for their area of exploration, and these partial solutions are gradually aggregated as the organisms fuse together. Finally, a single organism operates globally, building upon the partial solutions to form the overall solution to the problem. This approach presents a balance between speed and optimality by first using multiple organisms to explore the problem space in parallel and then letting a single organism globally optimize the result.

We dedicate the core of the paper Section “Results: explore-and-fuse approach” to present how the *explore-and-fuse* method can be used on the Euclidean Steiner tree problem of finding the shortest tree that connects a given set of points in a space, which is NP-hard. *Physarum Polycephalum* typically grows in moist forests and can be very large - up to several feet. Biological experiments have shown that *Physarum* can find shortest paths, solve mazes, form high-quality networks, share information through fusion, remember past events, and adapt to its environment^2,5,7^. In the present paper, we analyze the effect of cell shape and the number of cells on the algorithm behind our *explore-and-fuse* method before discussing the time complexity leading to the following findings:

- ***CELL shape*** Diamond CELLs give better solutions; Square CELLs are faster.
- ***CELL number*** Through a larger number of *Physarum* organisms in the swarm one can explore larger search areas, find better Steiner trees, find trees faster.

Applications of *Physarum* include drug repositioning, building unconventional computer chips, approximating highways, and designing subway systems^2,8–10^. In order to illustrate the novelty of the *explore-and-fuse* method as well as the benefits of its use, we dedicate Section “Discussion” to describing several different uses it has:

- ***Network design*** We use the algorithm to develop a road network in the United States and discuss characteristics which make it particularly suited to network design and other applications;
- ***Obstacle-avoidance*** We then use the algorithm to solve the obstacle-avoiding Euclidean Steiner tree problem and explain why the algorithm seems to be competitive with the current leading algorithm for this problem.
- ***Topological surfaces*** We discuss the algorithm’s adaptability to varying surfaces and boundaries by different considering topological (sphere, torus, Klein bottle, and $\mathbb{RP}\mathbb{}^2$).
- ***VLSI*** Finally, we use the algorithm to route a VLSI circuit board;

We conclude this paper discussing particularly noteworthy aspects of the algorithm as well as lines of further research in Section "Concluding remarks".

## Background

*Physarum Polycephalum* is an unicellular slime mold which is multi-nucleated and can be up to several feet large. It typically grows in moist forests and in the plasmodium stage of its life cycle, it forms many tubes. Cytoplasm streams through the tubes, changing directions every 1–3 min^11^. Moreover, *Physarum Polycephalum* is capable of learning and remembering despite being just a single-celled organism^5^. These organisms are also able to fuse and share information with each other as they fuse^12^. In what follows we shall first recall the CELL model, and then give a description of swarm algorithms and of the Steiner tree problem, keeping in mind the objective of this study, which is to introduce an *explore-and-fuse* model which is biologically-inspired and which could be used for solving large-scale problems that cannot be broken into independent sub problems

### CELL model

The CELL model, as described in^13^ and expanded in^6^, models a *Physarum* organism as a collection of squares on a grid. The key mechanism of this model is the rearrangement of cytoplasm and cytoskeleton (essentially the cell boundary) as external elements are introduced into the organism. Every square is assigned a state. A state of 0 represents a square that is not part of the organism, a state of 1 represents a piece of cytoplasm, and a state of 2 represents a piece of cytoskeleton. As the model runs, we update states using the rule that a square of cytoplasm in state 2 must neighbor at least one square of state 0 while a square in state 1 must not neighbor any square in state 0.

The model is defined by an algorithm which is repeated many times. At every step, a bubble, or piece of the outside (state 0), is introduced into the organism and slowly moves through it. By repeatedly moving squares of cytoplasm, the organism begins to move as a whole and take on different shapes.

The exact algorithm is as follows:
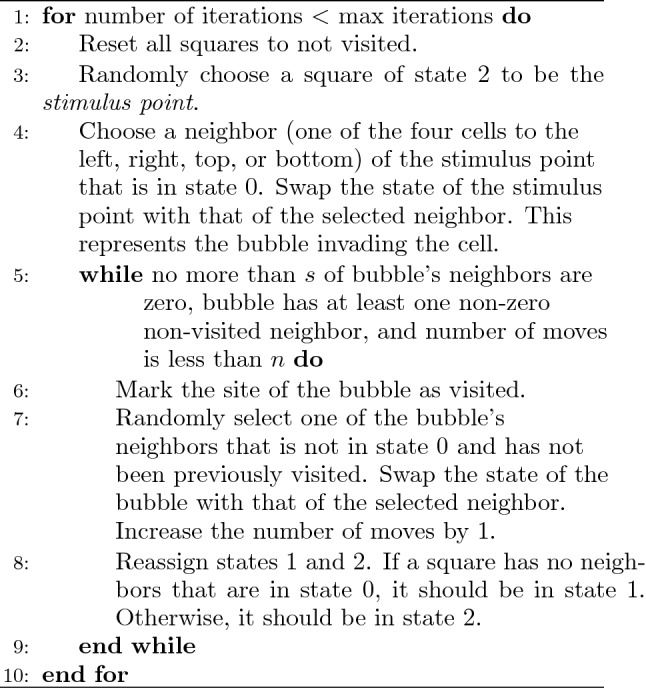


**The parameters and the stimulus points** In this model, there are two parameters to consider: *n* the number of swaps a bubble may take and *s* the maximum number of neighbors that can be in state 0. In this paper, all experiments are run with $n = 1000$ and $s = 3$. Depending on where stimulus points are selected from, the model exhibits different behavior. If stimulus points are randomly chosen, the cell behaves like an amoeba, randomly moving around. If stimulus points are always selected from certain regions called active zones, a network between the regions forms.

### Model of multiple cells

The CELL model^13^ is extended in^6^ to create the Model of Multiple Cells. In this model, one can spawn multiple cells which can be of different sizes. The main difference from the original CELL model is that stimulus points are randomly chosen from any square of state 2 across all cells. Once the cells fuse, or come in contact with each other, they are essentially treated as one cell. Bubbles can freely move between the two fused cells. Each cell is also given an ID which allows us to track which pieces of cytoplasm were originally from each cell as fusion occurs.

### Swarm algorithms

Particle swarm optimization (PSO) is an alternate approach to solving optimization problems that draws heavily on biological inspiration from organisms such as ants or bees. PSO consists of a swarm of agents who behave according to defined rules but collectively exhibit global behavior which can solve certain problems. For example, the movement of an agent may be determined according to a mathematical equation that takes into account the position of other agents. When the position (or other attribute) of many of these agents is taken into account, they collectively point to the optimal solution^14^. PSOs have been used to solve problems such as sequential ordering, assembly line balancing, protein-ligand docking, and DNA sequencing^15^. While *explore-and-fuse* has similarities to swarm algorithms, the key difference is that in *explore-and-fuse*, *Physarum* organisms are able to fuse and share knowledge. All *Physarum* organisms fuse into one large organism that takes into account local information and the global picture before creating the final solution.

### The Steiner tree problem

The Steiner tree has been a topic of great interest to mathematicians and computer scientists since the 19th century^16^. It has many practical applications including cable routing, chip design, drug repositioning, and phylogenetic tree routing^8,17–20^.

The general Steiner tree problem is to find the shortest tree that connects a set of given points (terminals) and can include additional points. There are many versions of the Steiner tree problem. The one most relevant to this work is the Euclidean Steiner tree problem. In this problem, the goal is to find the shortest tree between a set of points in a space. There is also a variation of the Euclidean Steiner tree problem which shall be of interest in the present paper: the obstacle avoiding Euclidean Steiner tree problem where the tree needs to avoid certain regions of the plane. The Euclidean Steiner tree problem is an NP-hard problem. In fact, even approximating the solution within a factor of 96/95 is NP-hard^21^.

Currently, there are multiple Steiner tree algorithms such as SCIP-Jack^22^, one developed by Polzin and Daneshmand^23^^24^, and GeoSteiner^25^. Each algorithm has different strengths and weaknesses so that depending on the characteristics of the graph and the variation of the Steiner tree problem, a different algorithm results in the best performance. SCIP-Jack is based on a combination of implications, conflicts, and reductions in a branch-and-cut framework. As the most recent algorithm, SCIP-Jack outperforms Polzin and Daneshmand’s algorithm on many benchmark sets and is able to solve test graphs with 100,000 vertexes in 477.9 s on average. Polzin and Daneshmand’s algorithm has long been considered state-of-art and at the time of its introduction outperformed existing algorithms by several magnitudes. GeoSteiner is a publicly available Steiner tree software first developed in 1985. GeoSteiner uses the approach of first generating full Steiner trees (FSTs) in phase 1 and then combining a subset of the FSTs to find a minimum Steiner tree in phase 2^26^.

There are also currently various *Physarum*-inspired Steiner tree algorithms, two of which we will describe briefly. The first algorithm^28^ was developed to solve the problem of relay node placement. This algorithm uses mathematical modeling similar to *Physarum* Solver^29^ of *Physarum*’s protoplasmic flow and tube thickening to create a system of equations that can be solved to determine the minimal tree. The second algorithm^8^ also uses a system of equations similar to *Physarum* Solver to solve the node weighted Steiner tree problem.

## Results: explore-and-fuse approach

In what follows we shall create the *Physarum Steiner algorithm*, which uses the model of multiple CELLs to introduce the *explore-and-fuse* approach. Considering *Physarum*’s skill at solving the traveling salesmen and Steiner tree problems plus its ability to share knowledge through fusion, *Physarum* is the perfect organism for *explore-and-fuse*. Multiple *Physarum* organisms can independently explore, quickly gaining local knowledge, and then this information can be shared via fusion, allowing for global optimization. This method of parallel exploration fusing into global optimization strikes a balance between speed and optimality.

**Figure 1:**
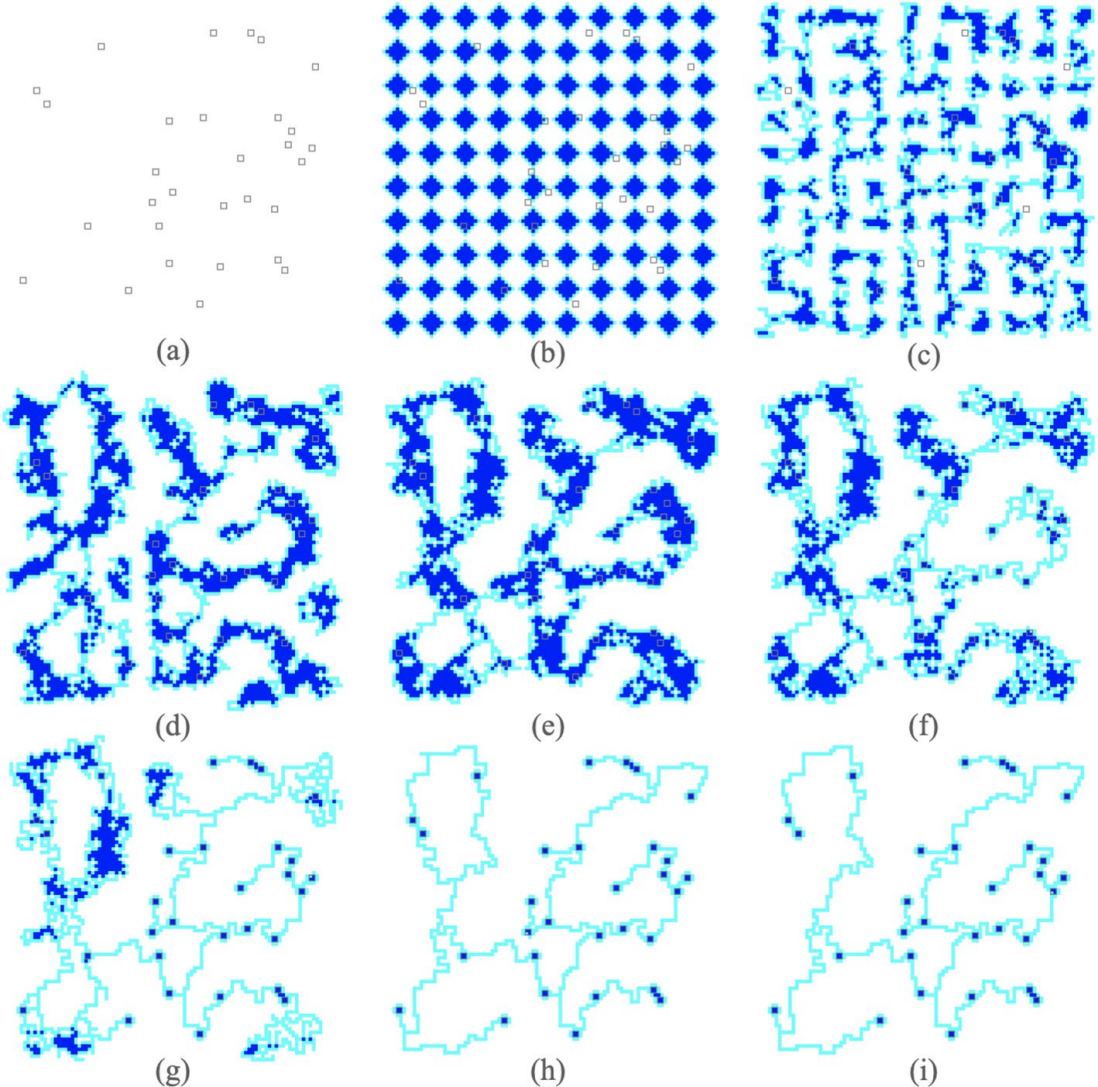
Progression of the algorithm: The starting grid with the points to be connected/active zones represented as $2 \times 2$ squares in (**a**). We begin by spawning 100 cells of size 7 in (**b**). In (**c**) and (**d**), the cells join and find points in the foraging phase. (**e**) marks the end of the foraging phase as one cell contains all the points. In (**f,g**), and (**h**) we see the removal of cytoplasm in the shrinking phase. Finally, in (**i**) we have the final solution.

The first step of this approach is to spawn multiple *Physarum* organisms as illustrated in Fig. 1b. Each organism then independently explores, partially solving portions of the problem. Organisms also come into contact with each other and fuse, beginning to share knowledge and combine partial solutions in Fig. 1c,d. By Fig. 1e, all of the *Physarum* organisms have fused into one large organism that has global knowledge. This organism can then begin to optimize the solution, taking into account all the local knowledge previously gathered, as seen in Fig. 1f–h. Finally, the solution is produced in Fig. 1i.

The *explore-and-fuse* approach can be seen as a less rigid form of divide-and-conquer. We believe that it can be applied to various problems such as the Steiner tree problem or traveling salesmen problem, and can inspire other ways to soften divide-and-conquer.

### Physarum Steiner algorithm

In this section, we demonstrate the *explore-and-fuse* approach on the Euclidean Steiner tree problem. The Euclidean Steiner tree problem is a good example to showcase the *explore-and-fuse* approach because it has many real-world applications such as electronic design and cable-laying, and is easy to visualize. Using the *explore-and-fuse* approach, we create the *Physarum Steiner Algorithm*, which uses the Model of Multiple Cells to generate approximate solutions to the Euclidean Steiner tree problem. An implementation of this algorithm has been made available at^31^.

This algorithm has two phases: first *foraging* where the cells find all points to be connected and then *shrinking* where the cell looses cytoplasm as it tries to find the minimum Steiner tree. We represent the points to be connected as $2 \times 2$ squares on a grid as shown in Fig. 1 (a). In this model, *N* is the number of points and *M* is the length of the square edge of the grid.

**Foraging** We utilize the fundamental mechanics of the Model of Multiple Cells, namely the movement of bubbles, and add a more complex selection of the stimulus point to cause the organisms to form a Steiner tree. Following the *explore-and-fuse* approach, we begin by spawning multiple *Physarum* organisms. Let $cells_{intial}$ be the number of cells initially spawned. We set each of the points to be connected as an active zone. On every iteration, we keep track of $points_{discovered}$, the number of active zones the cells are currently in contact with. We also track the number of disjoint cells which contain at least one point. We refer to this number as $cells_{effective}$. Note that if a cell does not contain a point, it is not counted in $cells_{effective}$.

Next, we repeatedly choose a random stimulus point to introduce the bubble and let it percolate through the cells. The stimulus point can be chosen from any square of cytoplasm in state 2; it is not limited to a certain cell. On every iteration, we have two options for the stimulus point. We can randomly choose a piece of cytoplasm in state 2 that lies inside any active zone that has already been found. This will bring cytoplasm to the active zone and help prevent the cells from moving away from active zones that have already been discovered. The other option is to randomly choose a square in state 2. This helps the cells explore in random directions and find more active zones. The probability $p_\text {random}$ that we choose the second option is defined according to:

1 $$\begin{aligned} p_\text {random}:= \frac{N - points_{discovered} + cells_{effective} - 1}{N + cells_{initial}}. \end{aligned}$$

In Eq. 1, the number of points not found is represented by $$N - points_{discovered} $$, and $$cells_{effective} - 1$$ represents the number of cells that still need to fuse. Consequently, the probability of choosing the second option that favors exploration is higher when there are more points left to find and more cells to be fused. When $$p_\text {random}$$ becomes zero (one organism is connecting all the points), it is time to move from the *foraging* phase of the algorithm to the *shrinking* phase.

**Shrinking** In this phase of the algorithm, $p_\text {random}$ is zero and thus stimulus points are selected in the active zones. When there are no stimulus points in the active zones, we randomly choose a piece of cytoplasm to remove from inside an active zone. We change the state of that square to zero, decreasing the area of the cell and also creating some viable stimulus points. We also keep track of the number of iterations since the area of the cell last changed. When this number passes a threshold (1 million was used in this paper), the algorithm terminates. While this termination approach does not necessarily guarantee a Steiner tree, we find that for the vast majority of applications such as network design, a couple extra loops or unnecessary connections are not problematic. If the final result absolutely must be a Steiner tree, it is possible to implement a floodfill algorithm to check if there are still loops in the tree and continue to run the shrinkage phase until there are no longer any loops.

### Time complexity

In this section, we analyze the time complexity of the *Physarum Steiner Algorithm*. There are two variables to be considered: *N* the number of points and *M* the size of the grid. We first analyze the effect of independently varying *N* and *M*, and then vary *N* at a fixed ratio to *M*. We measure the number of iterations that the algorithm takes to terminate. Note that each iteration of the *Physarum Steiner Algorithm* is not necessarily linear, but this is dependent on the specifics of the implementation which is beyond the scope of this paper. For example, if each cell is implemented using disjoint-set union data structure or checking for merges is only called on neighboring cells, the time complexity is greatly increased. We believe that there are still improvements that can be made to our implementation, which is publicly available^31^. For our time complexity trials, we use size 9 square cells spaced one apart which leads to a short foraging phase and a much longer shrinking phase.

**Figure 2:**
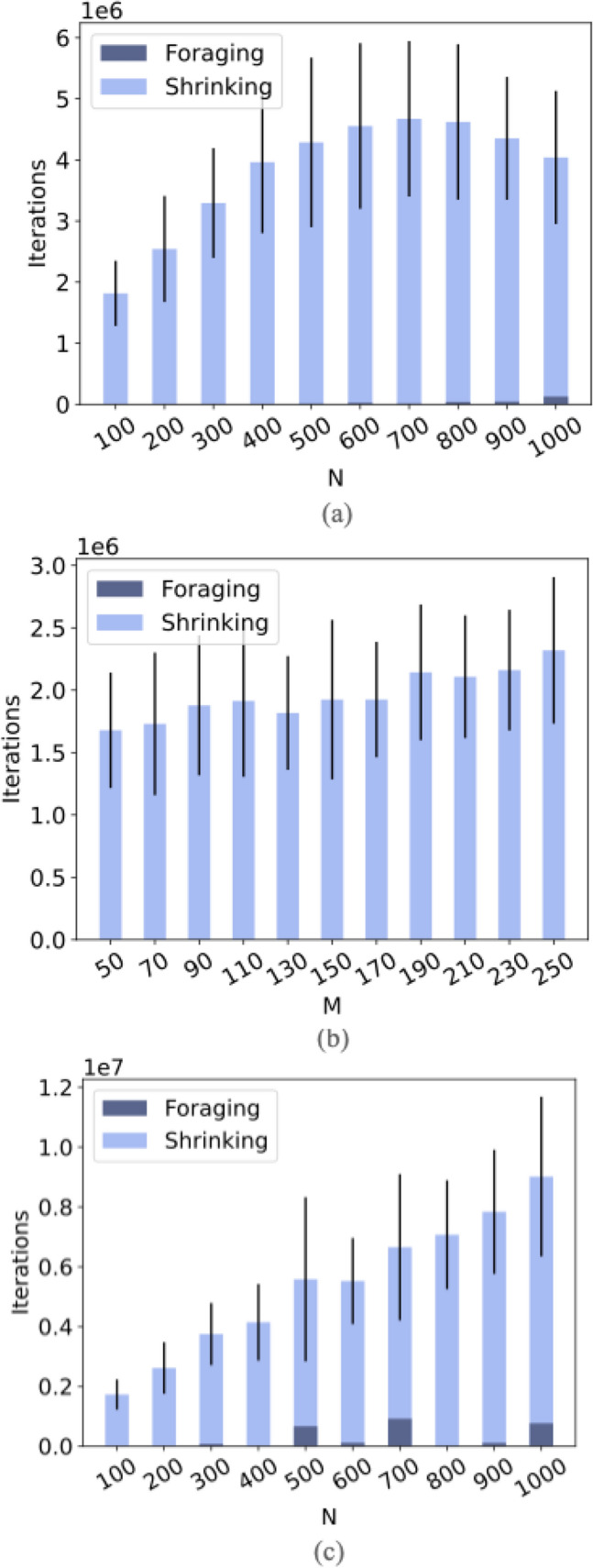
(**a**) Number of iterations varying with *N*. (**a**) Number of iterations varying with *M*. (**b**) Number of iterations varying with *N* normalized to 2 percent of $M \times M$. (**c**) Black lines on bars represents error (standard deviation). Failed trials excluded from graphs shown.

**Number of points** We first analyze the time complexity in terms of *N*, the number of points. We set *M* to be constant at 100. For every value of *N* from 100 to 1000, we generate 10 random $100 \times 100$ graphs. We run 10 trials on each of the graphs, for a total of 100 trials for each value of *N*. The algorithm has a very high success rate for finding a Steiner tree. Out of 1000 trials, only one failed to complete within 10 million iterations. This failed trial is excluded from the graph in Fig. 2a. In Fig. 2a, we see that the number of iterations appears to initially increase before decreasing. We hypothesize that the number of iterations decreases for larger values of *N* because as *N* increases, the final solution gets longer and thus there is not as much cytoplasm that needs to be removed through shrinkage. In addition, because there are more points, pieces of cytoplasm are more likely to be close to a point. Since cytoplasm is removed at points, or in other words bubbles are propagated from the points, if cytoplasm is closer to points there is a higher probability that it will be removed. Empirically, the time complexity of this algorithm appears to be less than linear in *N*. This is noteworthy considering the run time and time complexity of other Steiner tree algorithms.

**Grid size** We now analyze the time complexity in terms of *M*, the size of the grid. We set *N* to 100 while *M* takes on values from 50 to 250. We can think of *M* as a control for the resolution of the solution. As before, we run 10 trials on 10 graphs for every value of *M*. All trials of this experiment are successful. In Fig. 2b, we see that the number of iterations appears to increase very slowly with *M*. The gradual slope (less than $10^6$ iterations for a 200 unit increase in *M*) suggests that this algorithm scales well to larger search areas.

**Normalization** Finally, we consider what happens when *N* varies at a fixed ratio to *M*. We set *N* to always be two percent of the search area, or $M \times M$. We run trials where *N* takes values from 100 to 1000 and *M* is computed according to Eq. (2).

2 $$\begin{aligned} M =\left[ \sqrt{\frac{N}{0.02}} \right] \end{aligned}$$

As before, we run 10 trials on 10 graphs for each value of *N*. All but 4 of 1000 trials are successful. In Fig. 2c, we observe that the number of iterations increases linearly with *N* normalized to 2 percent of $M \times M$. We see some values of *N* with a considerable amount of time spent foraging. This may be due to the random generation of grids resulting in grids with points that are concentrated in out of the way locations. In summary, the empirical results presented in this section suggest that the algorithm will scale well to large problems.

**Figure 3:**
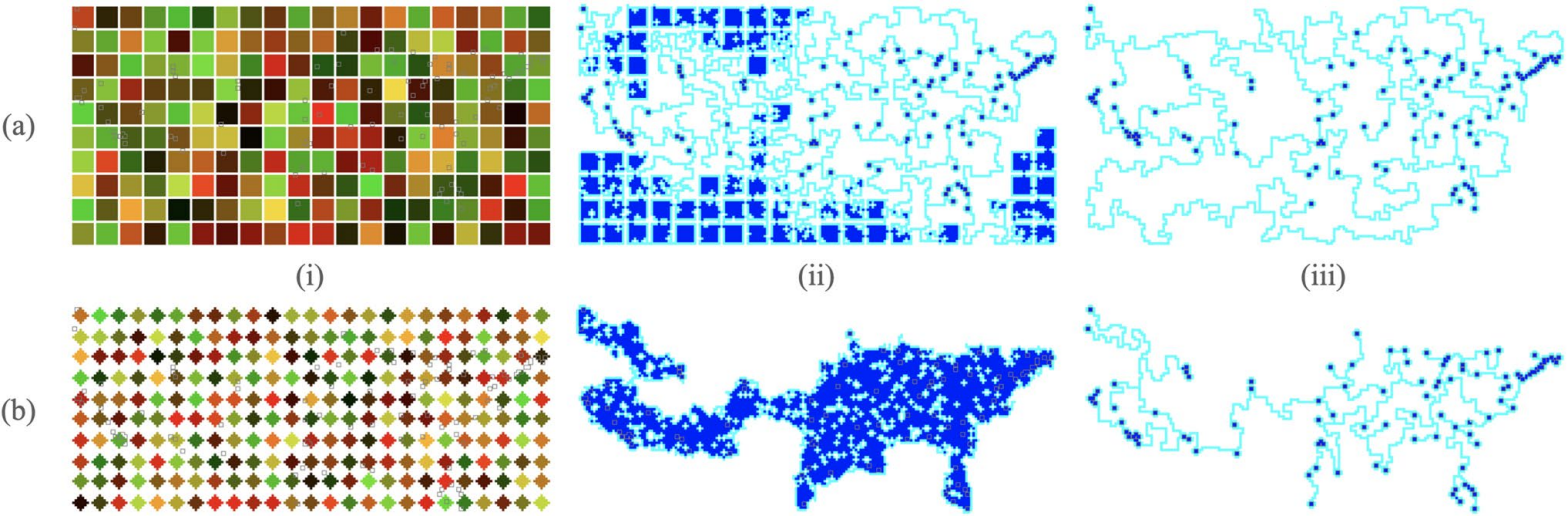
(**a**) Size 9 squares spawned one apart. Bottom image is final solution. (**b**) Size 7 diamonds spawned one apart. Bottom image is final solution.

## Discussion

Having introduced our novel *explore-and-fuse* method and the *Physarum Steiner Algorithm* we shall dedicate this section to discussing how the algorithm’s parameters influence the model, and how the method can be used towards diverse applications.

In what follows we shall consider how different parameters such as the different shapes of cells, as well as their number, influence the results obtained by the *Physarum Steiner Algorithm*. We shall then conclude the section by studying different applications that our methods have.

### Cell shape

Although^13^ and^6^ considered diamond shaped CELLs, we shall consider here CELLs with other shapes. The primary benefit of square cells is that their shape allows for more cytoplasm to be placed on the grid. As a result, the foraging phase is very fast so using square cells tends to result in shorter run times than using diamond-shaped cells. In addition, large square cells are able to more completely cover the standard square grid than diamond-shaped cells. On the other hand, diamond-shaped cells result in less cytoplasm and more time spent in the foraging stage. This gives the cytoplasm time to move towards a centralized location which results in better solutions.

***Example A*** In order to illustrate the above point, in Fig. 3a.i., we begin with squares that are tightly packed. Since the squares are so tightly packed (1 apart), if any piece of cytoplasm in a square is moved, it will lead to a connection with a neighboring cell. As a result, all the points are found very quickly. In fact, many of the squares are connected and part of the network even if they are not close to any of the points, as shown in Fig. 3 (a.ii.). Shrinking these extra squares takes a long time and can also result in long paths which are far out of the way as seen in Fig. 3a.iii.

***Example B*** In contrast to Example A, in Fig. 3b, we consider diamond-shaped cells. The cells start off diamond-shaped and with less overall cytoplasm than the square cells. The cells then spend quite a few iterations in the *foraging* phase. Although this does take time, it allows the cytoplasm to move towards a centralized location around the active zones as seen in Fig. 3 (b.ii.). When the cell finally proceeds to the *shrinking* phase, there is less cytoplasm to remove and no out of the way paths, resulting in shorter solutions. The downside to this is the increased time which in some cases can be very long (over 100 million iterations) and in some cases the algorithm may not even complete.

### The effect of multiple cells

In what follows we shall examine the effects of the number of cells used. We run 10 trials on 10 grids for a total of 100 trials on each cell size and number of cells. For each trial, we measure the total amount or area of cytoplasm that is initially spawned. This is used to normalize the search area which is the number of squares in the grid (for example a $100 \times 100$ grid has search area 10,000).

**Success rate:** The algorithm may sometimes be unsuccessful at connecting all the points. For example, the cells may miss a point early on and move far away from that point, making it almost impossible to ever find that point. There may also simply not be enough cytoplasm for two far away cells to fuse into one. For each number of cells (1, 9, 25, 100), we try various sizes/amounts of cytoplasm and compute the proportion of trials (out of 100) that successfully terminate within 10 million iterations.

**Figure 4:**
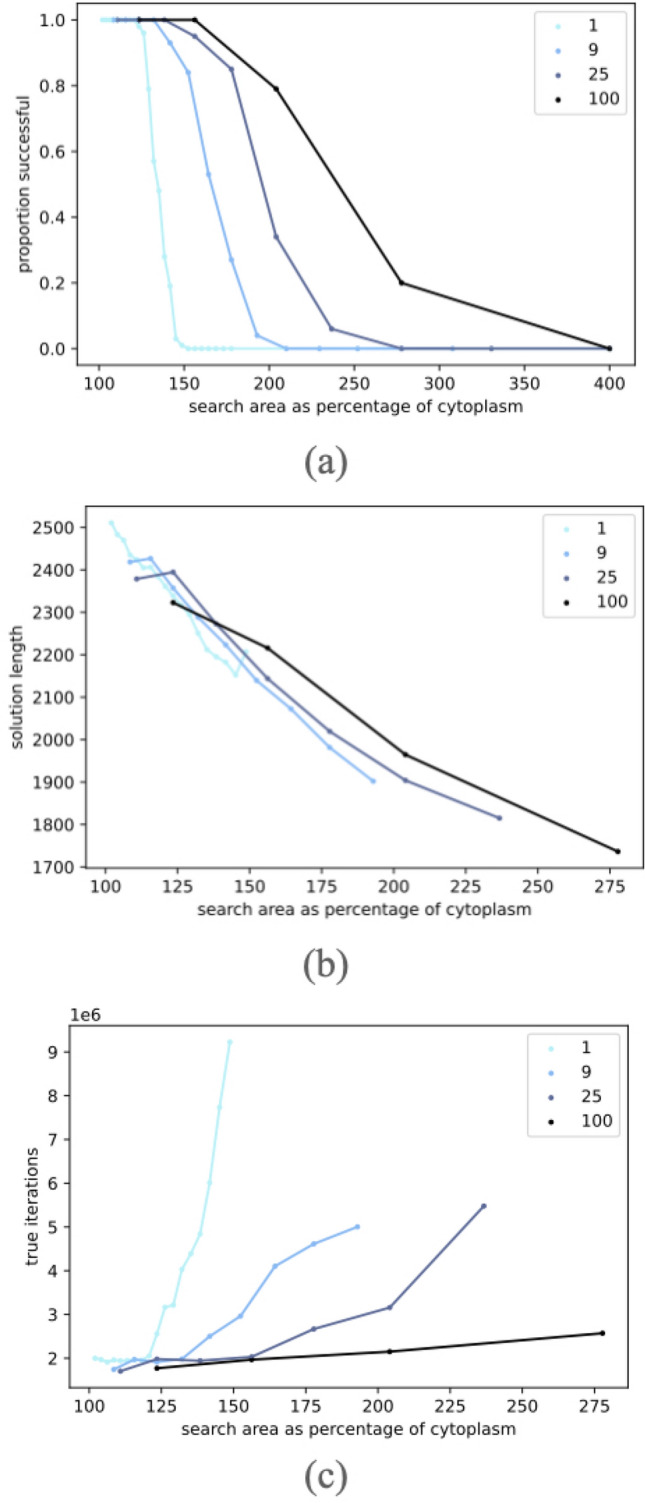
(**a**) Proportion of trials that are successful versus the search area as a percentage of cytoplasm for trials with 1, 9, 25, and 100 cells. (**b**) Length of solutions versus the search area as a percentage of cytoplasm. (**c**) Number of iterations versus the search area as a percentage of cytoplasm. Failed trails excluded from graphs.

In Fig. 4a, we see that the black line (100 cells) extends much further to the right than the cyan line (one cell). Thus, the more cells there are, the larger of a search area we can explore. This is mainly because with more cells, we can spread out our cytoplasm instead of having it be concentrated in certain areas.

**Solution length** Another important metric to consider is the solution length. We measure how good the solution is by counting the amount of cytoplasm when the algorithm terminates. We ignore any cytoplasm that is part of a disjoint cell that does not contain an active zone, or in other words is separate from the cell that actually forms the tree. In Fig. 4b, we see that as the search area as a percentage of cytoplasm increases, the quality of the solution improves. This is because there is comparatively less cytoplasm to begin with. In addition, we see that as the number of cells increases, it is possible to find a better solution. This correlates with the earlier result shown in Fig. 4a that using more cells allows solutions to be found with less cytoplasm. Trials with 100 cells found the shortest solutions (rightmost data point).

**Run time** The last metric we consider is the run time. We consider the true number of iterations the algorithm runs for. By true iterations, we account for the fact that in a parallel algorithm or set of real-world *Physarum* organisms, multiple cells will be introducing and moving bubbles at the same time. As a result, the iteration count is scaled by the number of disjoint cells. In Fig. 4c, we see that the more cells there are, the lower the number of iterations. This may be because with more cells, the cytoplasm is more spread out and therefore there are less out of the way points which may take a very long time to find. From the above analysis, we see that using more cells allows us to explore bigger search areas, find shorter solutions, and solve problems faster.

### Applications

The behavior of *Physarum* and the models it has inspired have found many different uses among which are drug repositioning, developing bio-computing chips, approximating highways layouts, and designing subway systems^2,8–10^. In order to illustrate the operation of the *Physarum Steiner Algorithm* and demonstrate its applicability to real world problems, we consider the following:

- sep0em
- ***Network design*** We use the algorithm to develop a road network in the United States.
- ***Obstacle-avoidance*** We use the algorithm to solve the obstacle-avoiding Euclidean Steiner tree problem.
- ***VLSI routing*** We use the algorithm to route connections between pads in chip design.
- ***Topological surfaces*** We discuss the algorithm’s adaptability to varying surfaces and boundaries by considering topological surfaces such as the sphere, torus, Klein bottle, and $\mathbb{RP}\mathbb{}^2$.

**Road networks** The *Physarum Steiner Algorithm* can be used to build a road network between the largest one hundred cities in the lower 48 United States (excluding Alaska and Hawaii). We use data^32^ containing the longitude and latitude of the 100 cities with the highest population to generate a rectangular grid of active zones.

**Figure 5:**
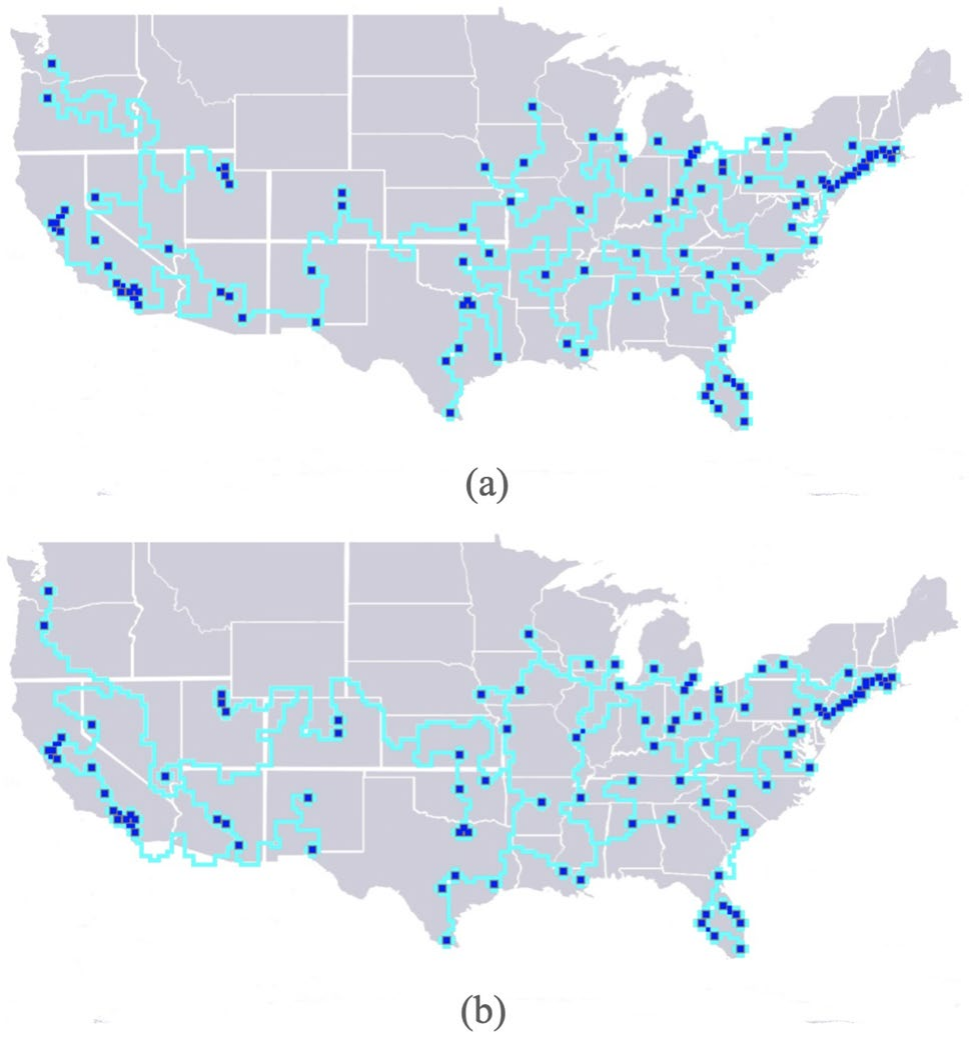
Road network generated by the algorithm. (**a**) shows the final solution with no loops while (**b**) displays a solution that has some redundancy resulting from terminating the algorithm early.

We spawn diamond-shaped cells of size 7 with a spacing of 1 as shown in Fig. 3. After many iterations, the final road network is shown in Fig. 5a. The algorithm is particularly suited to the problem of designing transportation systems because it first connects all the points before optimizing the network into a tree. The algorithm can thus be terminated early depending on how much redundant connectivity is desired in the transportation network.

For example, in Fig. 5b, we have a network that still contains loops in high-traffic routes between the Bay Area, Los Angeles, and Las Vegas. If we allow the algorithm to continue running, we will get networks with fewer loops and eventually a tree.

We believe that this algorithm can be applied to many similar problems such as designing fiber optic or electric cable networks. Moreover, as discussed in the last section, it will be very interesting to compare this study to that of^33^, where in vitro slime mold is used to investigate the construction of transportation networks over a USA map.

**Obstacle avoidance** Due to the cellular automaton nature of this algorithm, it is straightforward to define boundaries or other obstacles that need to be avoided. This is very useful in cases where certain areas need to be avoided such as a lake or the boundary of a county. And, unlike the current standard obstacle-avoiding Euclidean Steiner algorithm^27^ which takes multiple hours for graphs with only 150 points, the run time of the *Physarum Steiner Algorithm* is not affected by the need to avoid obstacles.

**Figure 6:**
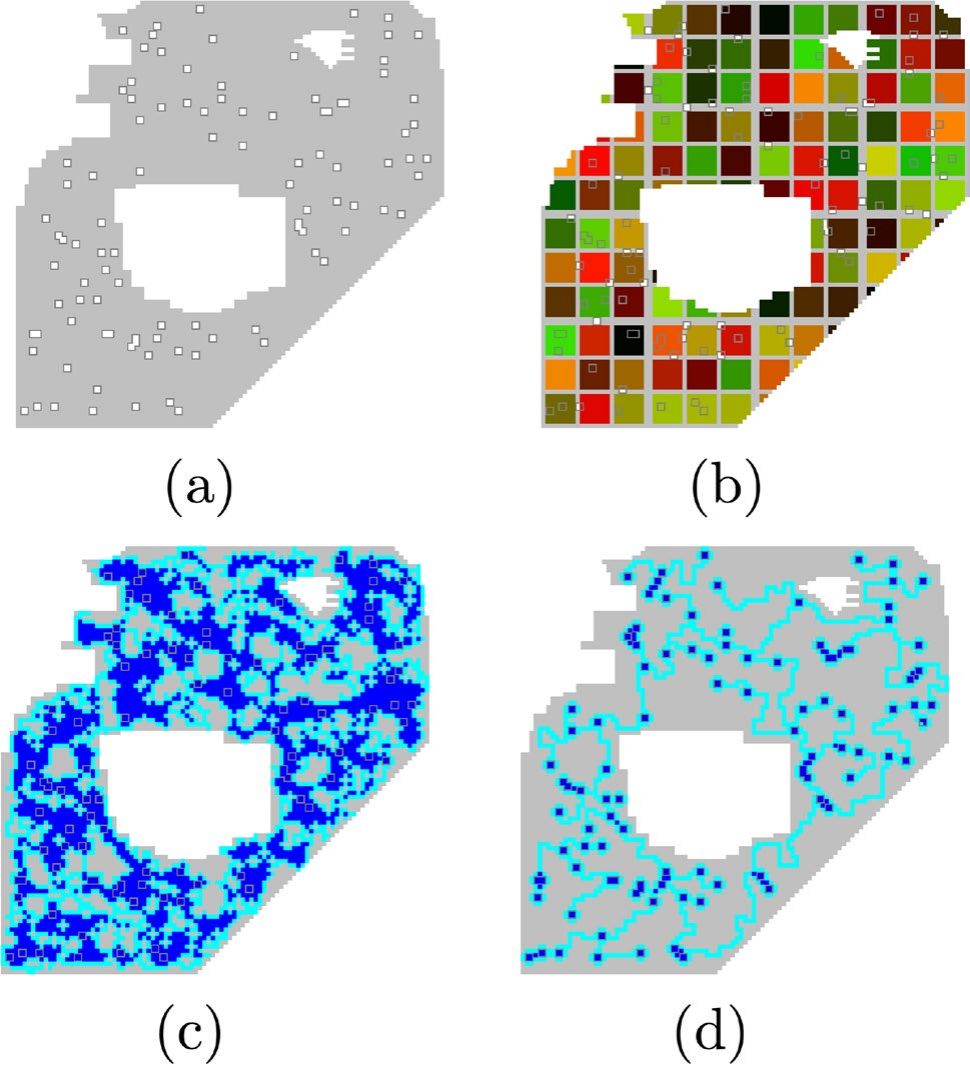
**(a)** Sample boundary map. Grey area is search area and small white squares are points. **(b)** Initial deployment of *Physarum*. **(c)** Solution at the end of the foraging stage. **(d)** The final network.

As an example, consider the boundary given in Fig. 6a. Here, the grey area represents the search area and the 100 white squares outlined in dark grey are the points. There are many possible real world situations similar to this. For example, the grey area could be a county and all the points represent homes that subscribe to a certain Internet service provider (ISP). The big white area in the center could be a lake and the smaller white area could be a dog park. The ISP company could utilize the *Physarum Steiner Algorithm* to find networks to lay fiber optic cables.

We begin by deploying square *Physarum* cells of size 7 in Fig. 6b. In Fig. 6c, the cells begin to fuse, share intelligence, and find all the points. We choose a solution that still has some loops to increase reliability and ease of future modification to the network. Our final solution is shown in Fig. 6d. This solution is generated in 300,000 iterations and less than 30 seconds.

**Figure 7:**
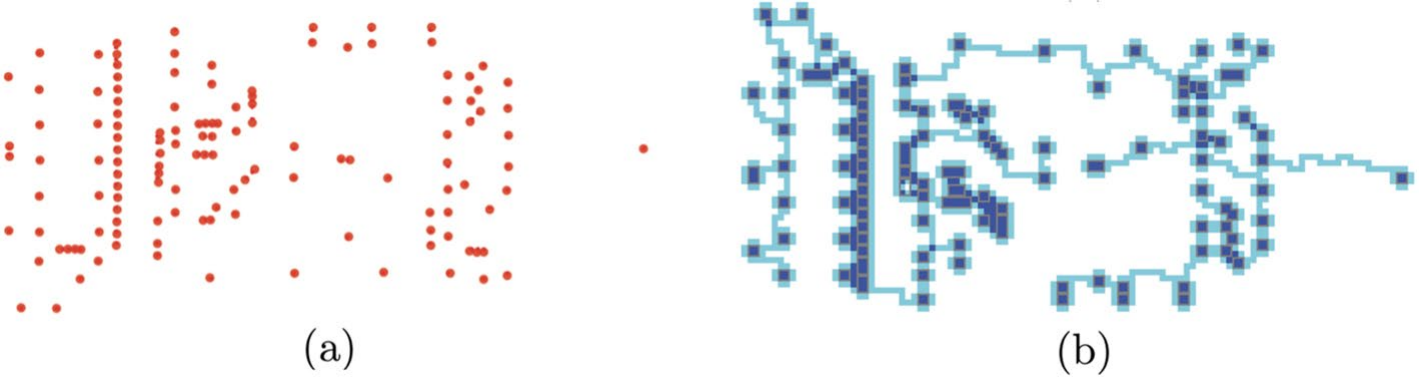
**(a)** Graphical representation of 131-point VLSI data set^34^. **(b)** Routing solution obtained by the *Physarum Steiner Algorithm*.

**VLSI** Routing for VLSI (very large-scale integration) chip design^19^ is one of the largest real-world manifestations of the Steiner tree problem, especially as modern chips may contain upwards of 10 billion transistors. Solving the VLSI problem would require additional modification to the *Physarum Steiner Algorithm* since VLSI design is typically presented as a group Steiner tree problem and has very large problem sizes, the *Physarum Steiner Algorithm. * Due to the usage of a square grid in the *Physarum Steiner Algorithm*, the algorithm is easily applied to find rectilinear networks such as those required for routing chips. In addition, our empirical results suggest that it should scale well to the large problem sizes common in chip design. Using data from^34^, we consider a set of pads that need to be connected. In Fig. 7, we represent the pads as active zones and generate a tree between them.

**Figure 8:**
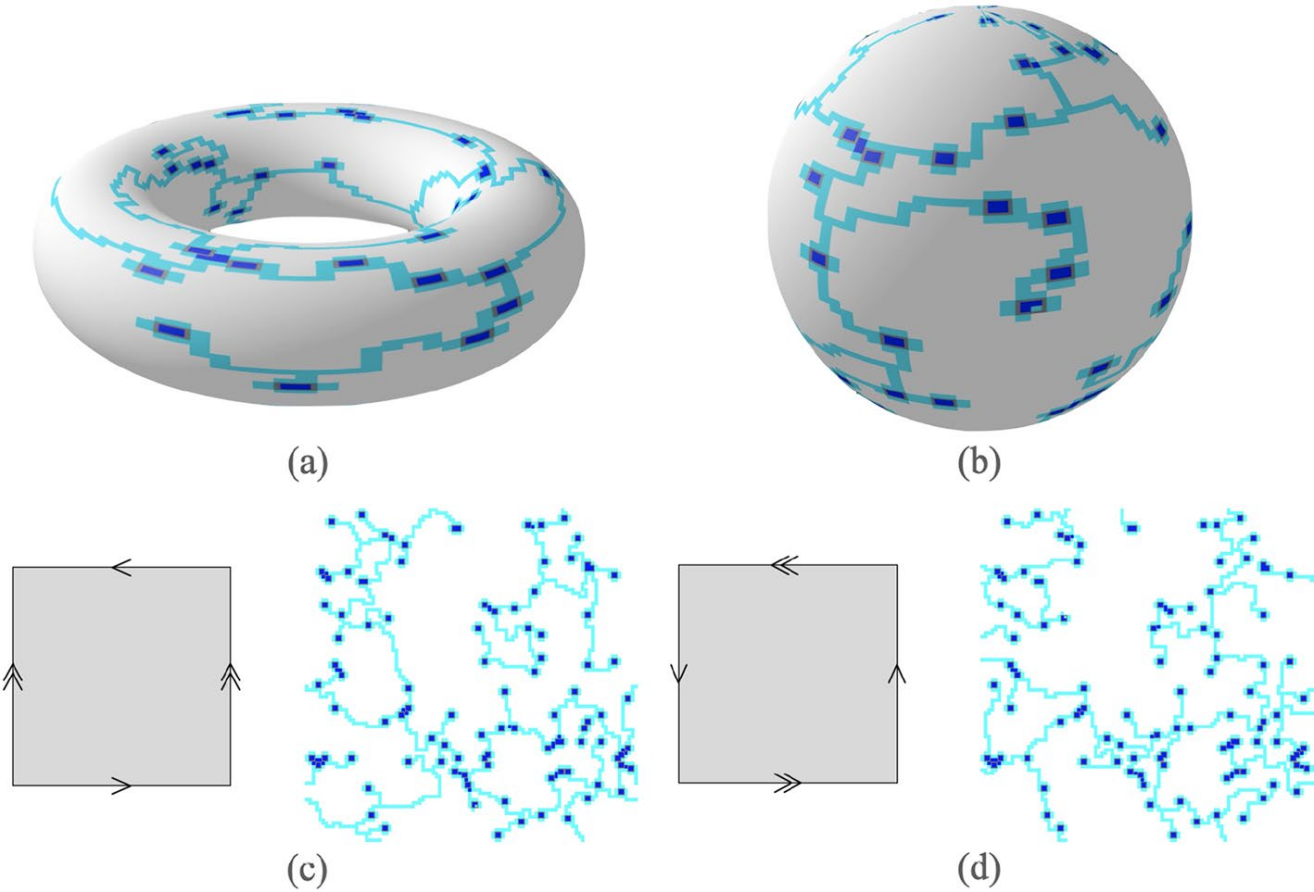
Steiner trees on topological surfaces we defined by identification space and obtained through our code. (**a**) Torus. (**b**) Sphere. (**c**) Klein Bottle. (**d**) $\mathbb{RP}\mathbb{}^2$. Images generated using manim^35^.

**Topological surfaces** Finally, the *Physarum Steiner Algorithm* is easily applicable to finding Steiner trees on other topological surfaces. Given the nature of the algorithm, we are able to map coordinates on one edge to another. In Fig. 8, we use square identification spaces to find Steiner trees on the torus, sphere, Klein bottle, and $\mathbb{RP}\mathbb{}^2$. These solutions on identification spaces can be seen on a torus and a sphere in Fig. 8a,b.

### Concluding remarks

We have presented here a novel *explore-and-fuse* approach to solve problems that cannot be solved by traditional divide-and-conquer.

Our approach is inspired by *Physarum*, a unicellular slime mold capable of solving the traveling salesman and Steiner tree problems. Besides exhibiting individual intelligence, *Physarum* can also share information with other *Physarum* organisms through fusion. These characteristics of *Physarum* inspire us to spawn many *Physarum* organisms to independently explore the problem space and collect information in parallel before sharing the information with other organisms through fusion. Eventually, all the organisms fuse into one large *Physarum* that can then globally optimize using the knowledge collected earlier. *Explore-and-fuse* can be seen as a less rigid form of divide-and-conquer that can better handle problems that cannot be decomposed into independent subproblems.

We demonstrate the explore-and-fuse approach on the Steiner tree problem by creating the *Physarum Steiner Algorithm*. This algorithm has the ability to incrementally find Steiner trees. The first solution tends to contain many loops that are removed with additional iterations of the algorithm. This incremental improvement is particularly useful for applications such as road and cable networks where some degree of redundancy in the connectivity is desired. In particular, it will be very interesting to compare our work to the the one done in^33^ where a protoplasmic network created by in vivo *Physarum* is considered to study and asses show the slime mold imitates the United States Interstate System. We foresee several applications of our algorithm in this direction, leading to similar findings to those appearing in the studies done in^33^.

The algorithm operates on a rectilinear grid and is particularly applicable to rectilinear Steiner tree problems such as those that often arise in VLSI design. In addition, the algorithm performs well on the obstacle-avoidance Euclidean Steiner tree problem.

In comparison to the existing *Physarum*-inspired Steiner tree algorithms described in Section “The Steiner tree problem”, the *Physarum* Steiner Algorithm uses a completely different mechanism. While the existing algorithms use a system of equations modeling the thickening of tubes as protoplasm flows through them, the *Physarum* Steiner Algorithm is based on modeling *Physarum* spatially moving around a grid and finding a tree between squares of the grid. In addition, it should be noted that the approach taking in existing algorithms would not work on the Euclidean Steiner tree problem as in the Euclidean Steiner tree problem, there are an infinite number of possible points that could be part of the Steiner tree (essentially any point in the plane). It would not be possible to write a system of equations representing the infinite possible points and edges. In the future, we believe further work could be done to improve the *Physarum* Steiner Algorithm. Since the *Physarum* Steiner Algorithm is an approximate algorithm, future improvements could be made so its approximations are closer to the actual optimal solution. In addition, it would be interesting to see this approach applied to other problems *Physarum* has been able to solve such as the traveling salesmen problem.
